# Parents’ Dental Knowledge and Oral Hygiene Habits in Slovak Children

**Published:** 2017-07

**Authors:** Anna NADAZDYOVA, Dagmara SIROTNAKOVA, Martin SAMOHYL

**Affiliations:** 1. Dept. of Stomatology and Maxilofacial Surgery, Faculty of Medicine, Comenius University, Bratislava, Slovak Republic; 2. Private Dental Clinic, Bratislava, Slovak Republic; 3. Institute of Hygiene, Faculty of Medicine, Comenius University, Bratislava, Slovak Republic

## Dear Editor-in-Chief

Oral health is one of the most important elements of general health and quality of life (1). Worldwide, 60%–90% of schoolchildren have dental cavities, often leading to pain and discomfort (2). Today’s world is the lack of enough knowledge regarding oral health, which results in inappropriate hygiene behaviour (3).

The goal of the study was to serve as an analysis of parents’ knowledge about the hygiene habits of their children, with an aim to stress the importance of the parents’ influence on dental prevention, and to show these parents’ influence on the dental health of their children. The study sample was recruited from children (7 – 13 years) attending primary schools in Slovakia. Overall, we collected 152 completed questionnaires. The questionnaire was anonymous and respected privacy of those involved. The questionnaire was filled in by the parents or legal guardians of minors.

95.4% of parents stated that their children brush their teeth by themselves. This habit was most frequently stated concerning children older than 3 years (28.3%). For 91.0% of the children in the sample group, their parents brushed their teeth before the children started to brush them themselves. 7.9% of children had not had their teeth brushed at all before they started to brush them themselves and 0.7% of children were only checked by their parents as to whether their teeth were clean. The most frequent duration of teeth brushing was two to three minutes (45.6%). One parent stated that their child brushes their teeth until they are clean. 36.2% of children prefer brushing their teeth in circular motions, 17.8% of children brush their teeth with vertical motions, 9.2% of children employ horizontal movements when brushing their teeth and 33.6% combine several types of motion during brushing. It would be appropriate to eliminate horizontal techniques during teeth brushing and check the group of children that apply combined techniques and vertical motions as to whether the cleaning is satisfactory and whether it hurts soft teeth tissue in particular. 58.6% of children apply the toothbrush on the teeth as well as the gums, and 32.2% of children apply the toothbrush only on the teeth. 9.2% of parents were not able to answer questions about their children’s teeth cleaning technique. 42.8% of children use a soft toothbrush. 54.6% of children prefer an anatomically shaped toothbrush, and 19.1% of parents do not know what kind of shape a toothbrush should have. 64.5% of parents stated that a dentist, dental nurse or dental hygienist showed them a proper method and technique of teeth brushing; however, 35.5% of parents had never received any information of this kind. Besides a toothbrush, in their teeth brushing, children also use toothpaste (78.9%), mouthwash (56.6%) and dental floss (17.8%). 59.2% of children go to a preventive dental check-up twice a year and 30.9% of children once a year.

According to the Slovak legislature, based on public health insurance, dental check-ups for patients younger than 18 should takes place twice a calendar year. Medical procedures in relation to dental cavities are partly covered by public health insurance only if a patient has gone for a preventive dental check-up in the previous calendar year. The study results will be presented to parents who are responsible for the dental hygiene and oral health of their children. Results of the pilot study show that parents do not have clear information about the dental hygiene and oral health of children. This information is frequently presented to children during group preventive programmes. Parents usually make the decisions about dental hygiene, correct selection of dental tools, as well as frequency and correctness of teeth brushing, and that is why it is crucial that parents have correct information.

It should be considered whether it would be better for parents to attend the group preventive programmes that take place at elementary schools for pupils.
